# The sequence at Spike S1/S2 site enables cleavage by furin and phospho-regulation in SARS-CoV2 but not in SARS-CoV1 or MERS-CoV

**DOI:** 10.1038/s41598-020-74101-0

**Published:** 2020-10-09

**Authors:** Mihkel Örd, Ilona Faustova, Mart Loog

**Affiliations:** Institute of Technology, University of Tartu, 50411 Tartu, Estonia

**Keywords:** Biochemistry, Molecular biology

## Abstract

The Spike protein of the novel coronavirus SARS-CoV2 contains an insertion ^680^SPRRAR↓SV^687^ forming a cleavage motif RxxR for furin-like enzymes at the boundary of S1/S2 subunits. Cleavage at S1/S2 is important for efficient viral entry into target cells. The insertion is absent in other CoV-s of the same clade, including SARS-CoV1 that caused the 2003 outbreak. However, an analogous cleavage motif was present at S1/S2 of the Spike protein of the more distant Middle East Respiratory Syndrome coronavirus MERS-CoV. We show that a crucial third arginine at the left middle position, comprising a motif R**R**xR is required for furin recognition in vitro, while the general motif RxxR in common with MERS-CoV is not sufficient for cleavage. Further, we describe a surprising finding that the two serines at the edges of the insert **S**PRRAR↓**S**V can be efficiently phosphorylated by proline-directed and basophilic protein kinases. Both phosphorylations switch off furin’s ability to cleave the site. Although phospho-regulation of secreted proteins is still poorly understood, further studies, supported by a recent report of ten in vivo phosphorylated sites in the Spike protein of SARS-CoV2, could potentially uncover important novel regulatory mechanisms for SARS-CoV2.

## Introduction

The novel SARS-CoV2 coronavirus that has caused hundreds of thousands of deaths worldwide in 2020 has puzzled scientists with its high infectivity and severe consequences for infected organs^1,2^. One of the hypotheses that emerged immediately after the release of the SARS-CoV2 sequence in January^3^ was that a unique insertion in its Spike protein, predicted to carry a crucial cleavage site for furin protease, could be the key for enhancing zoonotic transmission and infectivity^4–6^.

**Figure 1:**
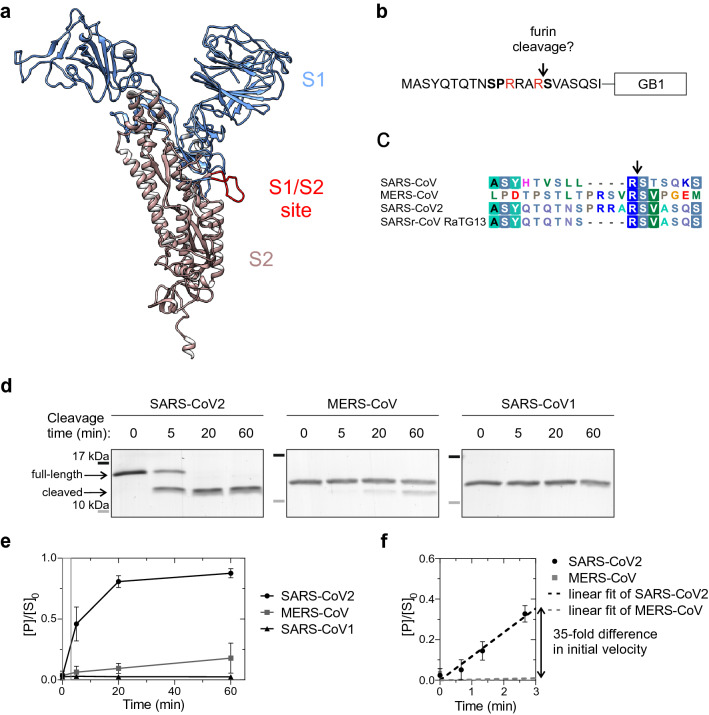
An insertion to the S1/S2 proteolytic cleavage site of SARS-CoV2 Spike protein introduces a furin site. (**a**) A structural model of SARS-CoV2 Spike protein^15^. Spike protein S1 (residue 1–685) is colored blue, Spike protein S2 (residue 686–1273) is colored brown and the intrinsically disordered S1/S2 proteolytic cleavage site is shown in red. The structure lacks the C-terminal residues 1148–1273. (**b**) Scheme of the constructs used to examine furin specificity. A 20-residue segment around the S1/S2 site was fused with a linker ELQGGGGG to the Streptococcal protein G B1 domain (GB1) and a C-terminal 6xHis tag. (**c**) Sequence alignment of the S1/S2 region in SARS-CoV, MERS-CoV, SARS-CoV2, and bat virus SARSr-CoV RaTG13, which is closely related to SARS-CoV2^16^. (**d**) Coomassie-stained SDS-PAGE gels showing the proteolytic cleavage of the GB1-fused reporter constructs. Furin activity towards the S1/S2 region of SARS-CoV2, MERS-CoV, and SARS-CoV1 was measured in vitro using the GB1 reporter constructs shown in ‘b’. The MW of SARS-CoV2 GB1 reporter protein is 10.9 kDa, which is cleaved by furin to 9.2 kDa and 1.7 kDa fragments. Uncropped images are shown in Supplementary Fig. S1. (**e**) Quantified data from the furin cleavage assay. The plot shows the relative amount of cleaved product compared to amount of uncleaved substrate at t = 0 min. Error bars show standard deviation. (**f**) Comparison of initial velocities of furin cleavage of SARS-CoV2 and MERS-CoV S1/S2 reporter constructs. To determine the initial velocity of SARS-CoV2 S1/S2 cleavage, the reaction was stopped at early time points (40 s, 1 min 20 s and 2 min 40 s) and these were used for the linear regression.

The coronavirus Spike glycoprotein mediates the entry of the coronavirus into the host cell^7^. It is composed of two subunits (Fig. 1a): S1, which binds the angiotensin-converting enzyme 2 (ACE2) on the host cell surface^8,9^, and S2, which mediates membrane fusion^10,11^. Proteolytic cleavage of the Spike protein by furin or other cellular proteases like TMPRSS2^8^ at the S1/S2 site is essential for the infection, as the cleavage separates two functions of Spike^12^. First, the S1 fragment will bind to the ACE2 receptor, and secondly, the S2 fragment will interact with the membrane^7^. Furthermore, coronavirus S proteins harbor another cleavage site located within the S2 domain, called the S2′, whose proteolysis is thought to expose hydrophobic side chains and trigger the membrane fusion^13,14^.

The 4-residue insertion underlined in the sequence ^674^YQTQTNSPRRAR↓SVASQ^690^ of the SARS-CoV2 Spike protein, where the arrow indicates the S1/S2 proteolytic cleavage site, contains a previously established cleavage motif RxxR↓x for furin^5,9^ (Fig. 1b). After the release of the SARS-CoV2 sequence, it was also promptly realized that an analogous R-x-x-R motif was present in the Middle East Respiratory Syndrome coronavirus MERS-CoV that caused an outbreak in 2012^4^. Strikingly, however, the predicted furin cleavage motif was missing in viruses belonging to the same clade as SARS-CoV2, including SARS-CoV1, the virus causing an outbreak in 2003^4^.

Importantly, the mechanism of proteolytic activation of Spike has been shown to play a major role in the selection of host species and the infectivity of coronaviruses^13,17^. Recently, it was reported that the cleavage of the SARS-CoV2 Spike at S1/S2 is required for viral entry into human lung cells^18^. However, the SARS-CoV2 spread also depends on cellular protease TMPRSS2^8,18^ and the direct role of cellular furin has remained undefined. It is also not yet known if the novel sequence with the R-x-x-R motif inserted at the S1/S2 site enables furin specificity and efficient furin-dependent cleavage. Furthermore, it has been reported that the MERS-CoV Spike, although also harboring an RxxR motif, is not activated directly by cellular furin during viral entry^19^. The question of Spike activation is extremely important to solve, since the initial mechanistic trigger of the current SARS-CoV2 pandemic could depend on the Spike cleavage site sequence as its host protease specificity can determine the zoonotic potential and “host-jump” of coronaviruses^13,17^.

Recent Cryo-EM structures of the SARS-CoV2 Spike protein revealed that the insertion with the furin-like cleavage site emerges as a disordered loop on the side of the protein^9,20^. Such intrinsically disordered regions (IDRs) are easily accessible for enzymes and other protein signaling modules, and often encode short linear motifs (SLiMs) that act as recognition sequences for such binding partners. Intriguingly, besides the RxxR motif, the sequence of the loop **SPRRA**R↓**S**V contains two serines that match the consensus of proline-directed kinases (SP), and basophilic protein kinases (RxxS), the two largest subfamilies of mammalian kinases^21,22^. However, the Spike protein, except for its short C-terminal tail, is considered to be facing the endoplasmic reticulum (ER) or the Golgi lumen during the viral replication cycle^23^ and is not present in cytoplasm or the nucleus, where most of the kinase signaling is taking place. Nevertheless, despite still being a poorly studied field, it is well known that protein phosphorylation does not only occur on cytoplasmic and nuclear proteins, but also takes place on secreted proteins in ER and Golgi lumen, and also in the extracellular space^24–27^. Strikingly, supporting evidence for the idea of Spike phospho-regulation arises from a recent report that confirmed more than ten in vivo phosphorylated sites in the Spike protein of SARS-CoV2^28^. Furthermore, phosphorylation has been shown to regulate fibroblast growth factor 23 targeting by furin^29^, indicating that kinases can regulate the secreted proteins as well as furin targeting.

In this study, we analyzed the furin cleavage site specificity of the SARS-CoV2 Spike using biochemical assays with substrate constructs based on the S1/S2 cleavage site sequence. We discovered that a motif RRxR, with a crucial P3 arginine (positions counted towards N terminus from the cleavage site, P4-P3-P2-P1), was required for cleavage of the S1/S2 site substrate constructs in vitro, while the motif in common with MERS-CoV (RxxR) was not sufficient for furin cleavage. Further, we describe a surprising finding that the two serines flanking the insert **S**PRRAR↓**S**V can be phosphorylated in vitro by different protein kinases and both phosphorylations affect the ability of furin to cleave the site. Finally, we discuss the possible novel regulatory mechanisms that such interplay of three different and mutually inter-dependent enzymatic modifications within the insert may present for SARS-CoV2 function.

## Results

### The furin cleavage consensus site is present in the S1/S2 site of Spike protein of SARS-CoV2 but not of MERS-CoV

As the furin cleavage site is in a disordered flexible loop at the side of the Spike protein^9,20^ (Fig. 1a), we set out to analyze the amino acid sequence specificity of furin cleavage using purified protein fragments corresponding to the disordered region and containing the S1/S2 cleavage site. The constructs were designed based on ^672^ASYQTQTNSPRRAR↓SVASQSI^692^ amino acids of Spike followed by a linker and a GB1-6xHis tag for purification (Fig. 1b). First, we created a set of substrate constructs based on the S1/S2 sequences of SARS-CoV2, MERS-CoV, and SARS-CoV1 (Fig. 1c). We followed the cleavage of the constructs by purified furin preparation in SDS-PAGE. Strikingly, we found that although the R-x-x-R motif at S1/S2 in MERS-CoV has been considered as a furin cleavage site^13,14,30^, it was cleaved by furin at very low efficiency (Fig. 1d,e). Contrarily, the novel coronavirus SARS-CoV2 site was cleaved very efficiently (Fig. 1d–f). Expectedly, the SARS-CoV1 motif lacking the furin site insertion showed no cleavage (Fig. 1d,e). As it is hard to determine the precise cleavage site using SDS-PAGE, we additionally performed a mass-spectrometric analysis of the cleaved fragments that confirmed the most abundant cleavage site was PRRAR↓SV in SARS-CoV2, and PRSVR↓SV in MERS-CoV constructs (Supplementary Table S1). N-terminal peptides that were cleaved from PRR↓ARSV and PR↓RARSV for SARS-CoV2, and PR↓SVRSV for MERS-CoV were detected with lower intensity, while the only forms of the C-terminal parts of the cleaved fragment started with an ↓SV-motif as predicted (Supplementary Table S1).

### P3 arginine in R-R-x-R motif is necessary for efficient cleavage of S1/S2 site substrate constructs by furin

**Figure 2:**
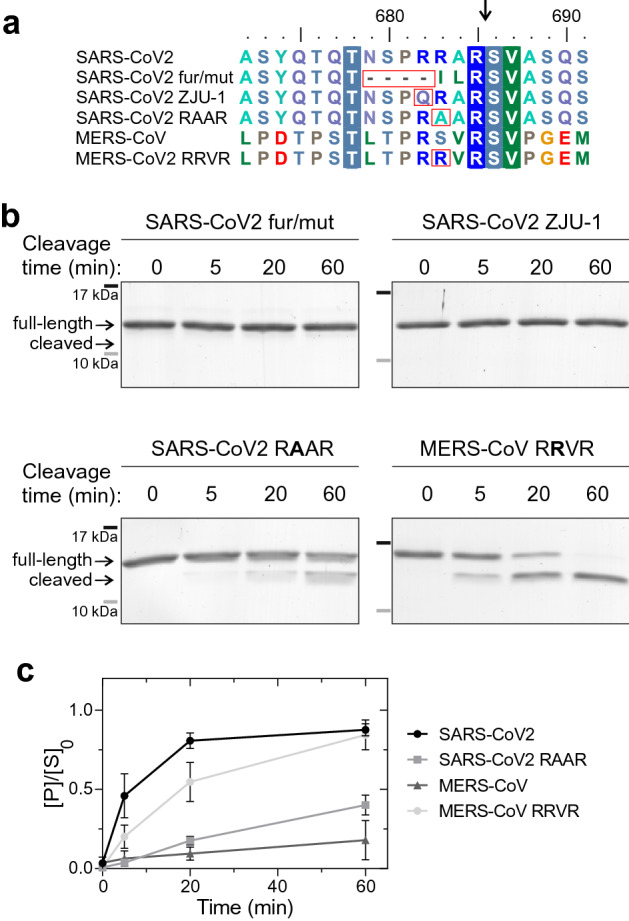
Analysis of furin cleavage efficiency of different SARS-CoV2 and MERS-CoV S1/S2 cleavage site mutants. (**a**) Alignment of SARS-CoV2 and MERS-CoV S1/S2 regions with the mutants used in substrate constructs for analysis of furin cleavage specificity in panel ’b’. (**b**) The furin cleavage efficiency of different mutants was analyzed in an in vitro time-course experiment. The proteolytic cleavage of S1/S2-GB1 reporter constructs by furin is visualized by Coomassie staining of SDS-PAGE gels. Uncropped images are shown in Supplementary Fig. S1. (**c**) The plot shows the accumulation of cleaved S1/S2-GB1 reporter protein in an in vitro furin activity assay. The error bars show standard deviation.

Next, we analyzed the furin cleavage of selected S1/S2 site mutants to better understand the specificity determinants of these sites. We used a recently described S1/S2 site mutant that lacks the four residue insertion (fur/mut^9^) (Fig. 2a) as a control to confirm the cleavage specificity in the in vitro furin assay. Deletion of the furin site in fur/mut-GB1 reporter protein abolished the proteolytic cleavage of the reporter protein (Fig. 2b). Next, a patient-derived mutation R682Q in Spike (ZJU-1^31^), that changes the furin core motif RxxR↓x to QxxR↓x, also completely inhibits furin activity towards the reporter protein (Fig. 2b). One difference between SARS-CoV2 and MERS-CoV S1/S2 sites is the presence of arginine 3 residues upstream of the cleavage site in the former (Fig. 2a). Interestingly, a mutation of this P3 residue to alanine (RRAR to RAAR) greatly decreased the furin cleavage rate compared to the wild-type SARS-CoV2 sequence (Fig. 2b,c). Introduction of arginine to the -3 position of MERS-CoV S1/S2 (RSVR to RRVR) considerably enhanced the cleavage rate by furin, resulting in only slightly lower cleavage efficiency compared to the wild-type SARS-CoV2 S1/S2 site (Fig. 2b,c). These findings highlight the presence of additional specificity determinants in the proteolytic cleavage sites on top of the commonly suggested RxxR, as a furin cleavage consensus motif. Further, this result shows how a single amino acid substitution can change protease specificity, which could have impacts on the tissue tropism and host range^32^. However, also the overall structuring of the region in and around the S1/S2 site, which is predicted to form a disordered loop in the case of SARS-CoV2, could depend on the context of native, full-length spike protein. This would affect accessibility of the sites and would impact how the Spike protein is recognized and processed by enzymes.

### The SARS-CoV2 S1/S2 cleavage motif could be phosphorylated by proline-directed and basophilic kinases

**Figure 3:**
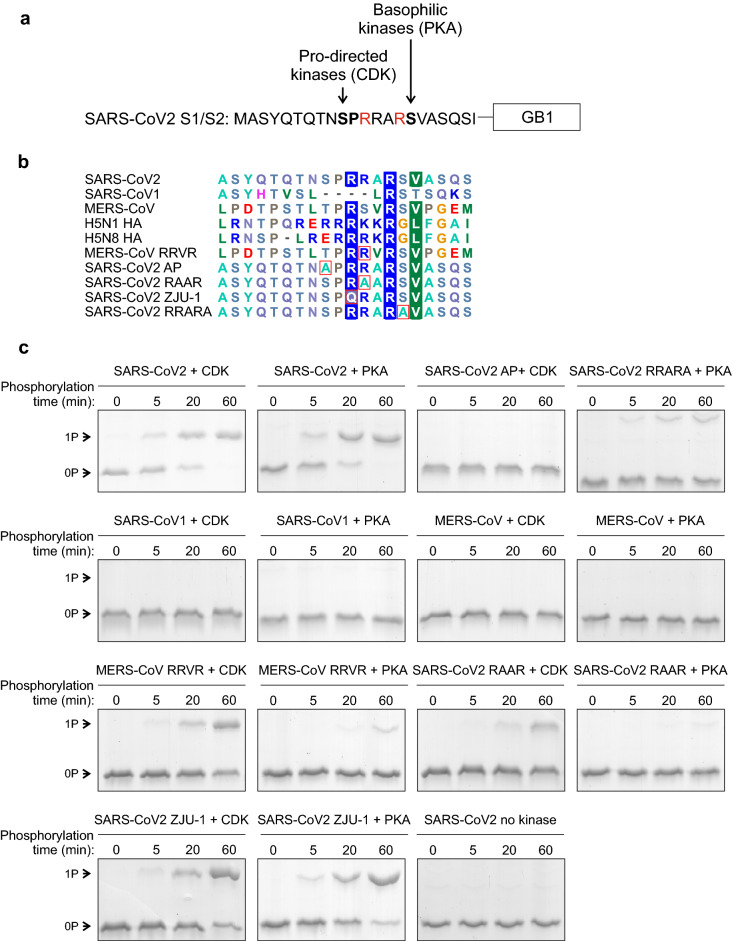
Potential phosphorylation of the S1/S2 site. (**a**) A scheme of the 20-residue segment of the SARS-CoV2 S1/S2 site. In addition to a furin site, the four-residue insertion (PRRA) creates potential phosphorylation sites for proline-directed kinases (S680) and basophilic kinases (S686). (**b**) Multiple sequence alignment of different S1/S2 sequences used in phosphorylation assays in panel ‘c’. (**c**) The phosphorylation of S1/S2-GB1 reporter proteins was analyzed in vitro using cyclin B-Cdk1 (CDK) and protein kinase A (PKA) as a representative of proline-directed and basophilic kinases, respectively. The phosphorylation reactions were stopped at 0, 5, 20, and 60 min. Phosphorylation shifts were analyzed using Phos-tag SDS-PAGE, which separates the phosphorylated form from the unphosphorylated form. Coomassie-stained gels are shown. Uncropped images are shown in Supplementary Fig. S3.

In addition to the furin cleavage site, the four amino acid insertion to the S1/S2 site of the SARS-CoV2 Spike protein also introduces potential phosphorylation sites that flank the core furin motif (Fig. 3a). Spike S680 matches the consensus of proline-directed kinases (SP) and S686 forms a consensus for basophilic kinases (RxxS), two large subfamilies of mammalian kinases^21,22^. Interestingly, the presence of potential phosphorylation sites can also be seen in the polybasic proteolytic cleavage sites of several other viral envelope proteins, including the ones of H5N1 and H5N8 influenza viruses (Fig. 3b). Importantly, while the phosphorylation of Spike proteins has not been studied thoroughly, several phosphorylated residues including both SP and RxxS sites have been identified in the SARS-CoV2 Spike protein by mass spectrometry^28^.

This prompted us to test whether the S1/S2 reporter proteins could be phosphorylated in vitro. For this, we used the cyclin B-Cdk1 complex (CDK) as a representative for proline-directed kinases and the protein kinase A (PKA) catalytic subunit as a representative for basophilic kinases. To examine the phosphorylation of different S1/S2-GB1 reporter proteins, we stopped the in vitro phosphorylation reactions at specific time points and analyzed the phosphorylation efficiency using Phos-tag SDS-PAGE to separate the phosphorylated protein from the non-phosphorylated substrate. The SARS-CoV2 S1/S2-GB1 reporter was fully phosphorylated by both CDK and PKA by the 60-min time point (Fig. 3c, Supplementary Fig. S2). Mutation of the predicted phosphorylation sites, S680 for CDK and S686 for PKA, abolished or greatly reduced the phosphorylation (Fig. 3c). The S1/S2 segments of SARS-CoV1 and MERS-CoV were expectedly not phosphorylated by PKA (Fig. 3c). The SARS-CoV1 S1/S2 segment does not contain a consensus site for proline-directed kinases, and while the MERS-CoV segment contains two potential proline-directed TP sites, these sites lack a basic residue in + 3 position, an important specificity determinant for CDK^33^. Introduction of the + 3R (denoted as P3 arginine for furin site) that greatly increases the furin cleavage efficiency of MERS-CoV S1/S2 also enhances its phosphorylation by CDK (Fig. 3c). Thus, the MERS-CoV S1/S2 segment could still be phosphorylated by other proline-directed kinases. Mutations in the + 2 and + 3 basic residues of SARS-CoV2 S680, which were found to affect furin cleavage (Fig. 2b), also decrease the phosphorylation rate by CDK (Fig. 3c), whereas with PKA, mutation of the -3R from S686 abolishes the phosphorylation, while mutation of -4R to Q has little effect (Fig. 3c).

### Phosphorylation inhibits furin cleavage of the S1/S2 site reporter proteins in vitro

**Figure 4:**
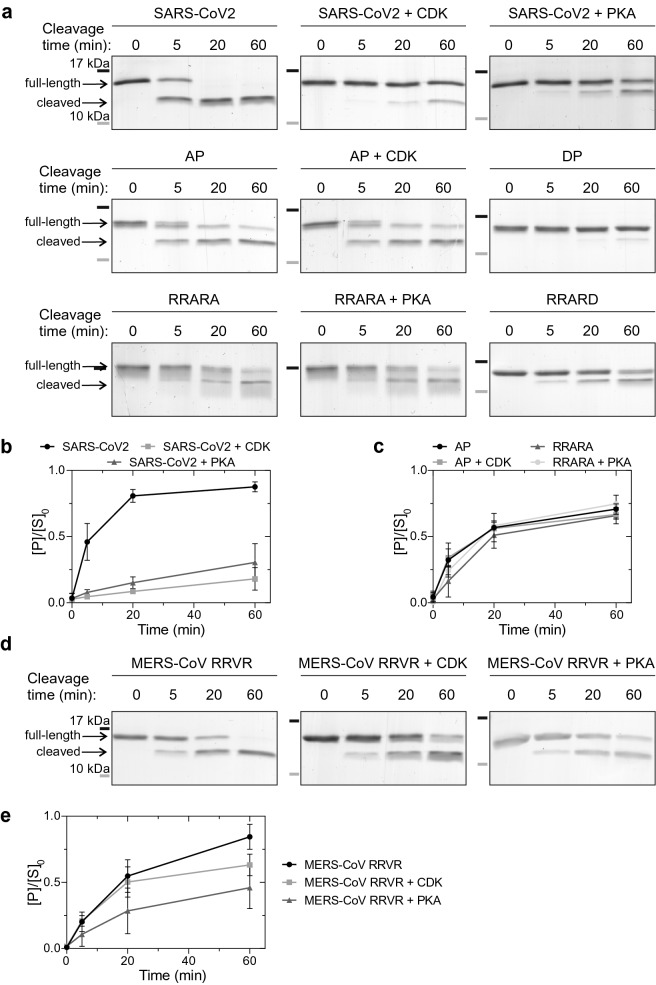
Phosphorylation at positions adjacent to the furin cleavage site inhibits the proteolytic cleavage in vitro. (**a**) The SARS-CoV2 S1/S2-GB1 reporter proteins were first phosphorylated with cyclin B-Cdk1 or PKA, followed by addition of furin. The furin activity was stopped at indicated time-points and the cleavage efficiency was analyzed by SDS-PAGE. (**b**) Plot showing the phosphorylation-dependent inhibition of furin activity on SARS-CoV2 S1/S2-GB1 reporter protein. Error bars show standard deviation. (**c**) Quantified furin cleavage profiles of the indicated SARS-CoV2 S1/S2-GB1 mutants without phosphorylation or with CDK- or PKA-mediated phosphorylation. Error bars show standard deviation. (**d**) Coomassie-stained SDS-PAGE gels showing the inhibitory effect of phosphorylation on furin-mediated cleavage of MERS-CoV S1/S2 with RRVR mutation. (**e**) Plot showing the relative abundance of cleaved MERS-CoV RRVR S1/S2-GB1 protein compared to the uncleaved form at t = 0. Error bars show standard deviation. Uncropped images are shown in Supplementary Fig. S4.

Next, we analyzed how phosphorylation at these sites affects furin cleavage. For this, the S1/S2-GB1 reporter proteins were first phosphorylated by either CDK or PKA for 60 min, resulting in the phosphorylation of the majority of the substrate, followed by the addition of furin. Phosphorylation of either site adjacent to the core furin motif, S680 and S686, significantly inhibited the furin cleavage (Fig. 4a,b). To confirm that the effect of phosphorylation is connected to the specific site, we analyzed the cleavage of alanine mutants of the phosphorylation sites. Interestingly, alanine mutations of these sites decreased the cleavage rate, although not to the same extent as phosphorylation, and the addition of kinase to the S680A and S686A substrates did not affect their cleavage further (Fig. 4a,c).

Next, we tested the effect of mutation of the phosphorylation sites to aspartic acid, often used to mimic phosphorylation. The S680D mutation decreased furin cleavage efficiency to the same extent as phosphorylation. However, S686D had a smaller effect on furin activity, similar to S686A mutation (Fig. 4a). Thus, the P + 1 site in the context of SARS-CoV-2 Spike protein requires the serine residue, and that other amino acids, not only the phosphorylation, whose side chains differ from serine can affect the cleavage, revealing a particular feature of SLiM R-x-x-R in the context of SARS-CoV2 Spike protein. Importantly, in a wider perspective, these results show that mutations and post-translational modifications outside the core RxxR furin motif significantly affect the cleavage.

A similar phospho-inhibitory effect was seen with MERS-CoV(RRVR)-GB1 (Fig. 4d,e), although the effect was less prominent, presumably due to less efficient phosphorylation of this protein (Fig. 3c), resulting in incomplete phosphorylation prior to furin addition. Taken together, these data reveal that phosphorylation adjacent to the furin motif can switch off the cleavage site.

## Discussion

Our results confirm that a four amino acid insertion to the S1/S2 site of the SARS-CoV2 Spike protein introduces a furin cleavage site. However, the P3 arginine, not present in analogous site in MERS-CoV, is crucial for furin-dependent in vitro cleavage of a substrate construct carrying the sequence of the S1/S2 site. While some previous reports have noted furin-mediated activation of MERS-CoV Spike^14,18^, others have argued against this due to an off-target effect of the furin inhibitor dec-RVKR-CMK^19^. Our finding suggests that SARS-CoV2 may have acquired a true furin cleavage specificity in contrast to the coronaviruses causing the two previous outbreaks. In addition, the discovered possibility of tight phospho-regulation at two serines creates an interesting complexity where a disordered loop carrying a SLiM facilitates specific regulatory inputs for three different modifying enzymes. The in vivo functionality of this SLiM could emerge as one of the key functional elements in the SARS-CoV2 Spike protein, given that proteolytic processing on viral glycoproteins has been found to be a key virulence factor. Indeed, previous studies have shown that highly pathogenic influenza virus forms have been found to harbor optimal polybasic furin processing sites, whereas forms with low pathogenicity have monobasic cleavage sites^34–36^. The glycoprotein cleavage specificity also determines tissue tropism, as furin is ubiquitously expressed, whereas the proteases that process monobasic cleavage sites, like TMPRSS2, are expressed mainly in the aerodigestive tract^37,38^.

The motif RxxR is often referred to as the minimal furin cleavage site, whereas RxK/RR forms an optimal motif that is cleaved efficiently^39,40^. More recent reports, however, have defined a core motif of 8 amino acids (6 residues N-terminal and 2 residues C-terminal of the cleavage site^41^. The work presented here also suggests that the furin motif is more defined and longer, as we find that RRxR is necessary for efficient cleavage of the tested S1/S2 sites by furin and that mutations flanking this core also affect the cleavage efficiency. Interestingly, the glycoprotein of highly pathogenic Ebolaviruses have been found to contain an optimal furin cleavage site, while the glycoprotein of a closely related Reston virus has a non-optimal cleavage site^42,43^. However, the requirement for P3 arginine for furin specificity discovered in this study suggests that different cellular proteases may prefer different variations of cleavage site sequences. One hypothesis would be that furin may present an activity and specificity common for different hosts and thus a window for zoonotic transfer.

Importantly, although not sufficiently studied, it is known that besides cyto- and nucleoplasm, protein phosphorylation occurs also in the extracellular space, and in lumens of ER and Golgi^24,25,44^. For example, one of the first discovered phosphoproteins was casein, a true secreted protein^45^. Recently, an analysis of the saliva phospho-proteome discovered close to a hundred phospho-proteins^46^. Thus, despite being a secreted protein, the Spike has a potential of being regulated by protein kinases. Moreover, a recent report presented in vivo evidence of more than ten phosphorylated sites at the Spike protein of SARS-CoV2^28^. The sites S680 or S686 were not detected in this analysis, presumably because of lack of coverage of this region in mass-spectrometry analysis. Further studies are required to understand if phosphorylation of the Spike protein has a physiological role and if specific kinases are involved.

In conclusion, the described short linear motif acting as a triple regulatory module is quite unique, also in a general signaling context. Further studies are required to establish its exact role in SARS-CoV2 and also as a modular regulatory element in vivo.

## Methods

### Protein purification

Constructs containing a 20 amino acid region from the S1/S2 linker were fused via a linker with the sequence ELQGGGGG to the GB1 domain (immunoglobulin-binding domain of streptococcal protein G) and a C-terminal 6xHis tag. The pET28a vectors were transformed to *E. coli* BL21 cells and protein expression was induced at 37 °C by addition of 1 mM IPTG. The His-tagged proteins were purified by cobalt affinity chromatography using Chelating Sepharose (GE Healthcare). The proteins were eluted in buffer containing 25 mM Hepes–KOH (pH 7.4), 300 mM NaCl, 10% glycerol, 200 mM imidazole.

### Phosphorylation assay

The phosphorylation of the S1/S2 linker constructs was examined in vitro using purified cyclin B-Cdk1 (Millipore 14-450) and PKA (murine cAMP-dependent protein kinase), purified as described in^47^. The phosphorylation reactions were carried out in buffer consisting of 50 mM Hepes–KOH (pH 7.4), 150 mM NaCl, 5 mM MgCl_2_, 50 mM imidazole, 2.5% glycerol, 0.2 mg/ml bovine serum albumin, 0.15 mM EGTA, 1 mM β-mercaptoethanol, and 500 μM ATP. The kinase reactions were performed in 30 µl containing 2.5 µg of S1/S2-GB1-6xHis substrate (7.6 µM). The concentration of PKA was 375 nM and cyclin B-Cdk1 5 nM. The reactions were carried out at room temperature and were stopped at 10 s (0 min), 5 min, 20 min, and 60 min by pipetting 5.5 µl of the reaction mixture to 8 µl of 2 × Laemmli SDS-PAGE sample buffer containing 1 mM MnCl_2_.

The stopped samples were incubated at 72 °C for 5 min and were loaded on Phos-tag SDS-PAGE gels containing 12.5% acrylamide, 100 µM Phos-tag, 200 µM MnCl_2_. The electrophoresis was carried out at 15 mA until the bromophenol blue dye front reached the bottom of the gel. Following electrophoresis, the gels were soaked in fixation solution (10% acetic acid, 30% ethanol aqueous solution) with gentle agitation for 15 min. The proteins were stained with colloidal Coomassie Blue G-250^48^.

### Furin cleavage assay

The furin cleavage specificity was assayed by incubating 2.5 µg (7.6 µM) S1/S2-GB1-6xHis substrate with 2 U furin (New England Biolabs, p8077) in 30 µl reactions. The reaction buffer contained 50 mM Hepes–KOH (pH 7.4), 150 mM NaCl, 5 mM MgCl_2_, 16 mM imidazole, 2% glycerol, 0.2 mg/ml bovine serum albumin, 0.15 mM EGTA, 500 μM ATP, 0.5% Triton-X100, 2 mM CaCl_2_, 2 mM β-mercaptoethanol. In case the effect of phosphorylation on furin cleavage was studied, the S1/S2-GB1-6xHis substrate was first phosphorylated for 60 min as described above, followed by addition of furin. The reactions were carried out at room temperature and were stopped at 0, 5, 20 and 60 min by pipetting 6 µl of the reaction mixture to 2 × Laemmli SDS-PAGE sample buffer.

The stopped reactions were heated at 72 °C for 5 min and loaded to 15% acrylamide SDS-PAGE. Following electrophoresis, the gels were immersed in fixation solution for 15 min and stained with colloidal Coomassie Blue G-250.

### Mass-spectrometric analysis of cleavage sites

Furin cleavage reactions were set up as described above, except that BSA was not added to the reactions. The reaction mixtures were incubated for 60 min at room temperature and were stopped by adding EGTA to 10 mM concentration to inhibit furin. Reactions where 10 mM EGTA was present at the time of furin addition were carried out for controls. The peptides arising from furin cleavage were purified using C18 StageTips. The peptides were separated using Agilent 1200 series nanoflow system (Agilent Technologies) and sprayed into an LTQ Orbitrap mass spectrometer (Thermo Electron) with a nanoelectrospray ion source (Proxeon). The data was analyzed using MaxQuant^49^.

## Supplementary information

Supplementary Figures.

Supplementary Tables.

## Data Availability

All data generated or analyzed during this study are included in this published article (and its Supplementary Information files).

## References

[1] Huang C (2020). Clinical features of patients infected with 2019 novel coronavirus in Wuhan, China. Lancet.

[2] Sanche S (2020). High contagiousness and rapid spread of severe acute respiratory syndrome coronavirus 2. Emerg. Infect. Dis..

[3] Chan JFW (2020). Genomic characterization of the 2019 novel human-pathogenic coronavirus isolated from a patient with atypical pneumonia after visiting Wuhan. Emerg. Microbes Infect..

[4] Coutard B (2020). The spike glycoprotein of the new coronavirus 2019-nCoV contains a furin-like cleavage site absent in CoV of the same clade. Antiviral Res..

[5] Andersen KG, Rambaut A, Lipkin WI, Holmes EC, Garry RF (2020). The proximal origin of SARS-CoV-2. Nat. Med..

[6] Cheng J (2019). The S2 subunit of QX-type infectious bronchitis coronavirus spike protein is an essential determinant of neurotropism. Viruses.

[7] Shang J (2020). Cell entry mechanisms of SARS-CoV-2. Proc. Natl. Acad. Sci. USA.

[8] Hoffmann M (2020). SARS-CoV-2 cell entry depends on ACE2 and TMPRSS2 and is blocked by a clinically proven protease inhibitor. Cell.

[9] Walls AC (2020). Structure, function, and antigenicity of the SARS-CoV-2 spike glycoprotein. Cell.

[10] Lu L (2014). Structure-based discovery of Middle East respiratory syndrome coronavirus fusion inhibitor. Nat. Commun..

[11] Du L (2009). The spike protein of SARS-CoV: a target for vaccine and therapeutic development. Nat. Rev. Microbiol..

[12] Heald-Sargent T, Gallagher T (2012). Ready, set, fuse! The coronavirus spike protein and acquisition of fusion competence. Viruses.

[13] Park JE (2016). Proteolytic processing of middle east respiratory syndrome coronavirus spikes expands virus tropism. Proc. Natl. Acad. Sci. USA..

[14] Kleine-Weber H, Elzayat MT, Hoffmann M, Pöhlmann S (2018). Functional analysis of potential cleavage sites in the MERS-coronavirus spike protein. Sci. Rep..

[15] Zhang C (2020). Protein structure and sequence reanalysis of 2019-nCoV genome refutes snakes as its intermediate host and the unique similarity between its spike protein insertions and HIV-1. J. Proteome Res..

[16] Zhou P (2020). A pneumonia outbreak associated with a new coronavirus of probable bat origin. Nature.

[17] Menachery VD (2020). Trypsin treatment unlocks barrier for zoonotic bat coronavirus infection. J. Virol..

[18] Hoffmann M, Kleine-Weber H, Pöhlmann S (2020). A multibasic cleavage site in the spike protein of SARS-CoV-2 is essential for infection of human lung cells. Mol. Cell.

[19] Matsuyama S (2018). Middle east respiratory syndrome coronavirus spike protein is not activated directly by cellular furin during viral entry into target cells. J. Virol..

[20] Wrapp D (2020). Cryo-EM structure of the 2019-nCoV spike in the prefusion conformation. Science.

[21] Hanks SK, Hunter T (1995). The eukaryotic protein kinase superfamily: kinase (catalytic) domain structure and classification. FASEB J..

[22] Miller CJ, Turk BE (2018). Homing in: mechanisms of substrate targeting by protein kinases. Trends Biochem. Sci..

[23] Lontok E, Corse E, Machamer CE (2004). Intracellular targeting signals contribute to localization of coronavirus spike proteins near the virus assembly site. J. Virol..

[24] Klement E, Medzihradszky KF (2017). Extracellular protein phosphorylation, the neglected side of the modification. Mol. Cell. Proteomics.

[25] Yalak G, Vogel V (2012). Extracellular phosphorylation and phosphorylated proteins: not just curiosities but physiologically important. Sci. Signal..

[26] Dartt DA, Hodges RR, Zoukhri D, Mircheff AK (1996). Protein phosphorylation in Golgi, endosomal, and endoplasmic reticulum membrane fractions of lacrimal gland. Curr. Eye Res..

[27] Preisinger C (2004). YSK1 is activated by the Golgi matrix protein GM130 and plays a role in cell migration through its substrate 14-3-3ζ. J. Cell Biol..

[28] Davidson AD (2020). Characterisation of the transcriptome and proteome of SARS-CoV-2 reveals a cell passage induced in-frame deletion of the furin-like cleavage site from the spike glycoprotein. Genome Med..

[29] Tagliabracci VS (2014). Dynamic regulation of FGF23 by Fam20C phosphorylation, GalNAc-T3 glycosylation, and furin proteolysis. Proc. Natl. Acad. Sci. USA.

[30] Millet JK, Whittaker GR (2014). Host cell entry of Middle East respiratory syndrome coronavirus after two-step, furin-mediated activation of the spike protein. Proc. Natl. Acad. Sci. USA.

[31] Yao H-P (2020). Patient-derived mutations impact pathogenicity of SARS-CoV-2. SSRN Electron. J..

[32] Millet JK, Whittaker GR (2015). Host cell proteases: critical determinants of coronavirus tropism and pathogenesis. Virus Res..

[33] Songyang Z (1994). Use of an oriented peptide library to determine the optimal substrates of protein kinases. Curr. Biol..

[34] Kido H (2012). Role of host cellular proteases in the pathogenesis of influenza and influenza-induced multiple organ failure. Biochim. Biophys. Acta.

[35] Sun X, Tse LV, Ferguson AD, Whittaker GR (2010). Modifications to the hemagglutinin cleavage site control the virulence of a neurotropic H1N1 influenza virus. J. Virol..

[36] Nao N (2017). Genetic predisposition to acquire a polybasic cleavage site for highly pathogenic avian influenza virus hemagglutinin. MBio.

[37] Thomas G (2002). Furin at the cutting edge: from protein traffic to embryogenesis and disease. Nat. Rev. Mol. Cell Biol..

[38] Lukassen S (2020). SARS-CoV-2 receptor ACE 2 and TMPRSS 2 are primarily expressed in bronchial transient secretory cells. EMBO J..

[39] Cao J, Rehemtulla A, Pavlaki M, Kozarekar P, Chiarelli C (2005). Furin directly cleaves proMMP-2 in the trans-golgi network resulting in a nonfunctioning proteinase. J. Biol. Chem..

[40] Krysan DJ, Rockwell NC, Fuller RS (1999). Quantitative characterization of furin specificity: energetics of substrate discrimination using an internally consistent set of hexapeptidyl methylcoumarinamides. J. Biol. Chem..

[41] Tian S, Huang Q, Fang Y, Wu J (2011). FurinDB: a database of 20-residue furin cleavage site motifs, substrates and their associated drugs. Int. J. Mol. Sci..

[42] Volchkov VE, Feldmann H, Volchkova VA, Klenk HD (1998). Processing of the Ebola virus glycoprotein by the proprotein convertase furin. Proc. Natl. Acad. Sci. USA..

[43] Braun E, Sauter D (2019). Furin-mediated protein processing in infectious diseases and cancer. Clin. Transl. Immunol..

[44] Tagliabracci VS, Pinna LA, Dixon JE (2013). Secreted protein kinases. Trends Biochem. Sci..

[45] Levene PA, Hill DW (1933). On a dipeptide phosphoric acid isolated from casein. J. Biol. Chem..

[46] Stone MD (2011). Large-scale phosphoproteomics analysis of whole saliva reveals a distinct phosphorylation pattern. J. Proteome Res..

[47] Kivi R, Loog M, Jemth P, Järv J (2013). Kinetics of acrylodan-labelled cAMP-dependent protein kinase catalytic subunit denaturation. Protein J..

[48] Candiano G (2004). Blue silver: a very sensitive colloidal Coomassie G-250 staining for proteome analysis. Electrophoresis.

[49] Cox J, Mann M (2008). MaxQuant enables high peptide identification rates, individualized ppb-range mass accuracies and proteome-wide protein quantification. Nat. Biotechnol..

