# Supplementary material for: The sequence at Spike S1/S2 site enables cleavage by furin and phospho-regulation in SARS-CoV2 but not in SARS-CoV1 or MERS-CoV
Source: Sci Rep. 2020 Oct 9;10:16944. doi: 10.1038/s41598-020-74101-0 (PMC7547067; doi:10.1038/s41598-020-74101-0)
Supplement: Supplementary file 1 — Supplementary Figures. [file 41598_2020_74101_MOESM1_ESM.pdf]

SUPPLEMENTARY INFORMATION

The sequence at Spike S1/S2 site enables cleavage by furin and phospho-regulation in SARS-CoV2 but not in SARS-CoV1 or MERS-CoV

Mihkel Örd, Ilona Faustova, and Mart Loog*

Institute of Technology, University of Tartu, Tartu 50411, Estonia

* Corresponding author (mart.loog@ut.ee)

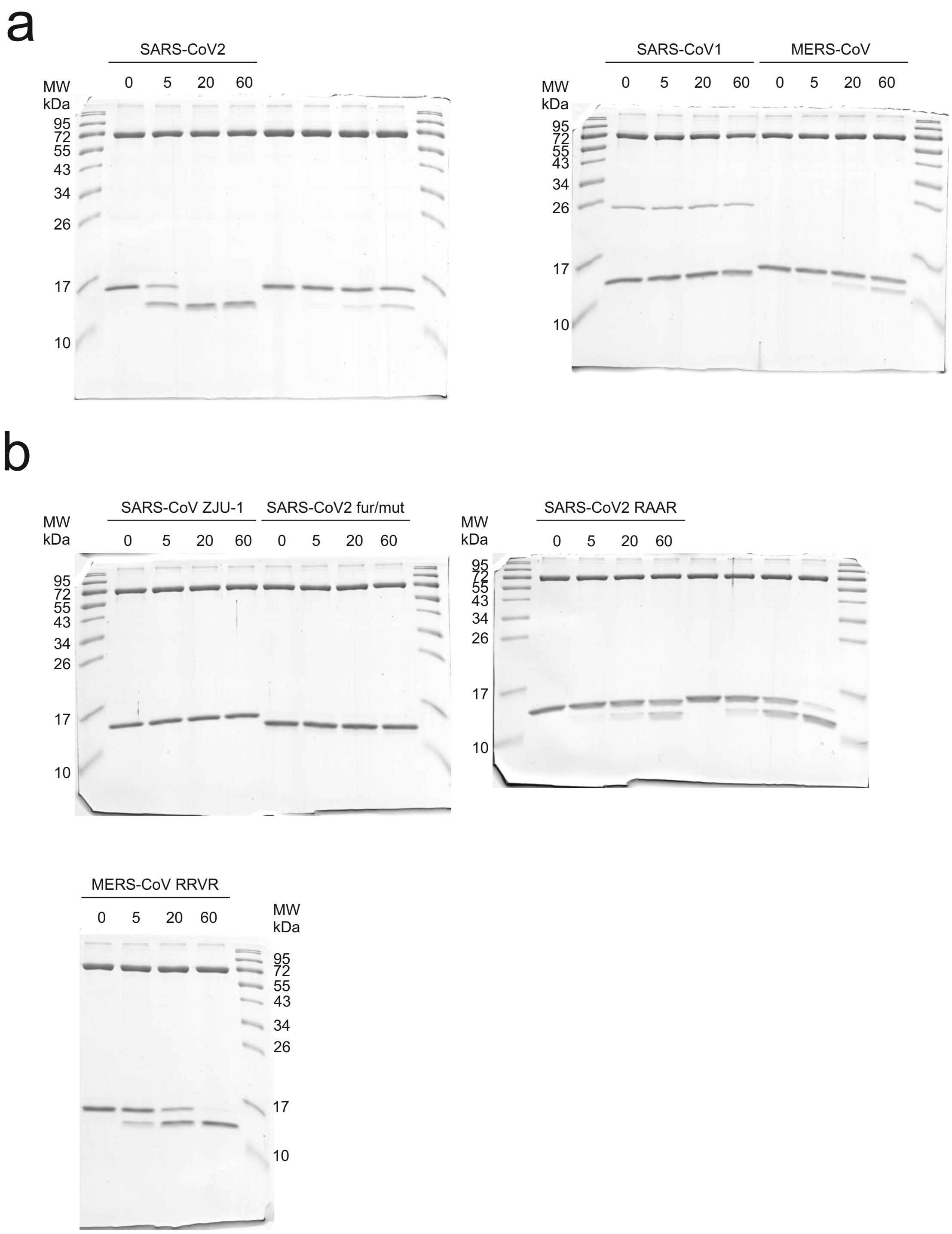

Supplementary Figure 1. Uncropped gels of Figure 1 (a) and Figure 2 (b).

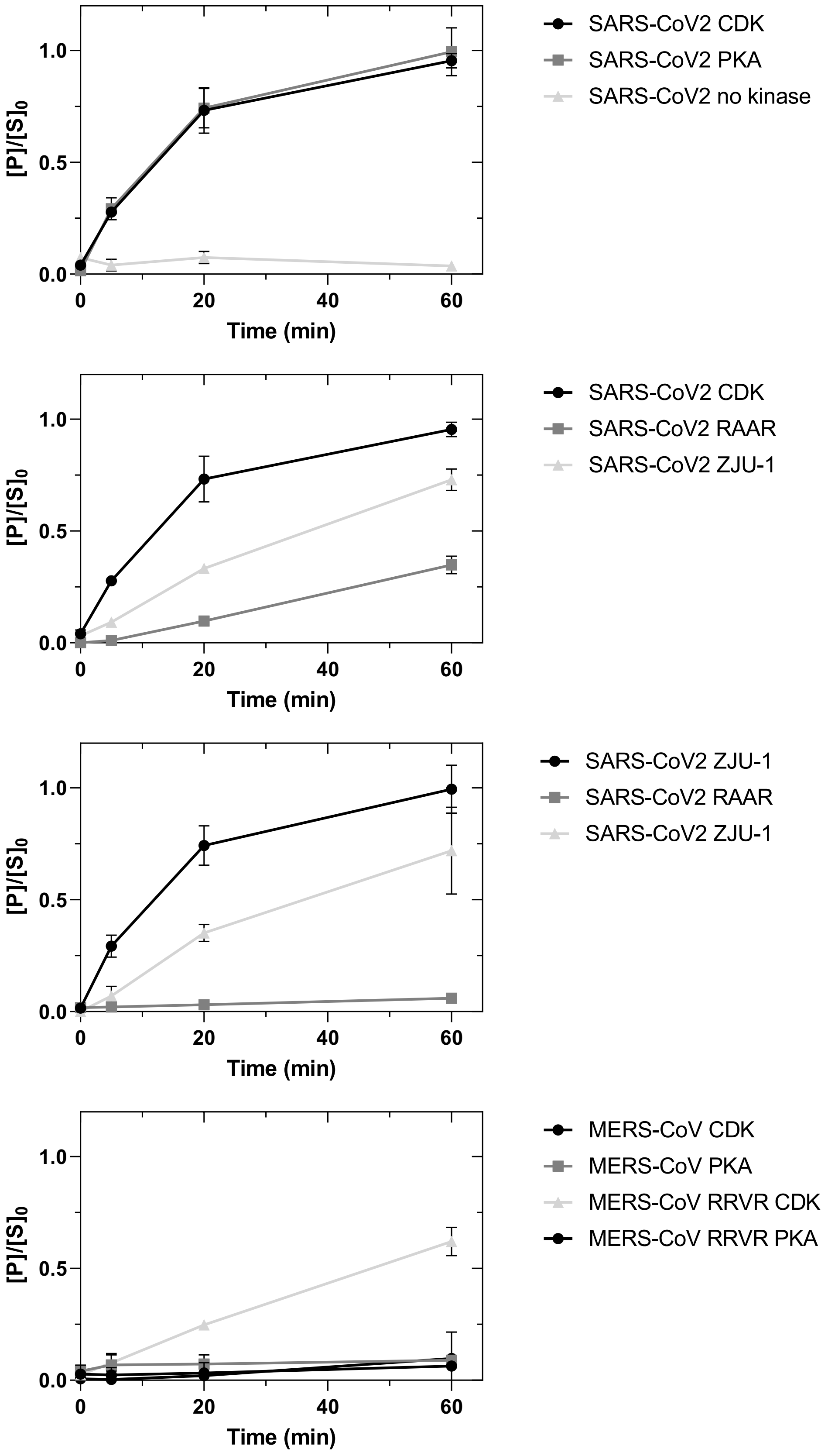

Supplementary Figure 2. Phosphorylation of SARS-CoV2 and MERS-CoV S1/S2 sites *in vitro*. The plots show accumulation of phosphorylated form of the indicated S1/S2-GB1 reporter protein in phosphorylation assays presented in Figure 3. The plots show relative abundance of phosphorylated form ([P]) compared to non-phosphorylated form at t=0 ($[S]_0$). Error bars show standard deviation.

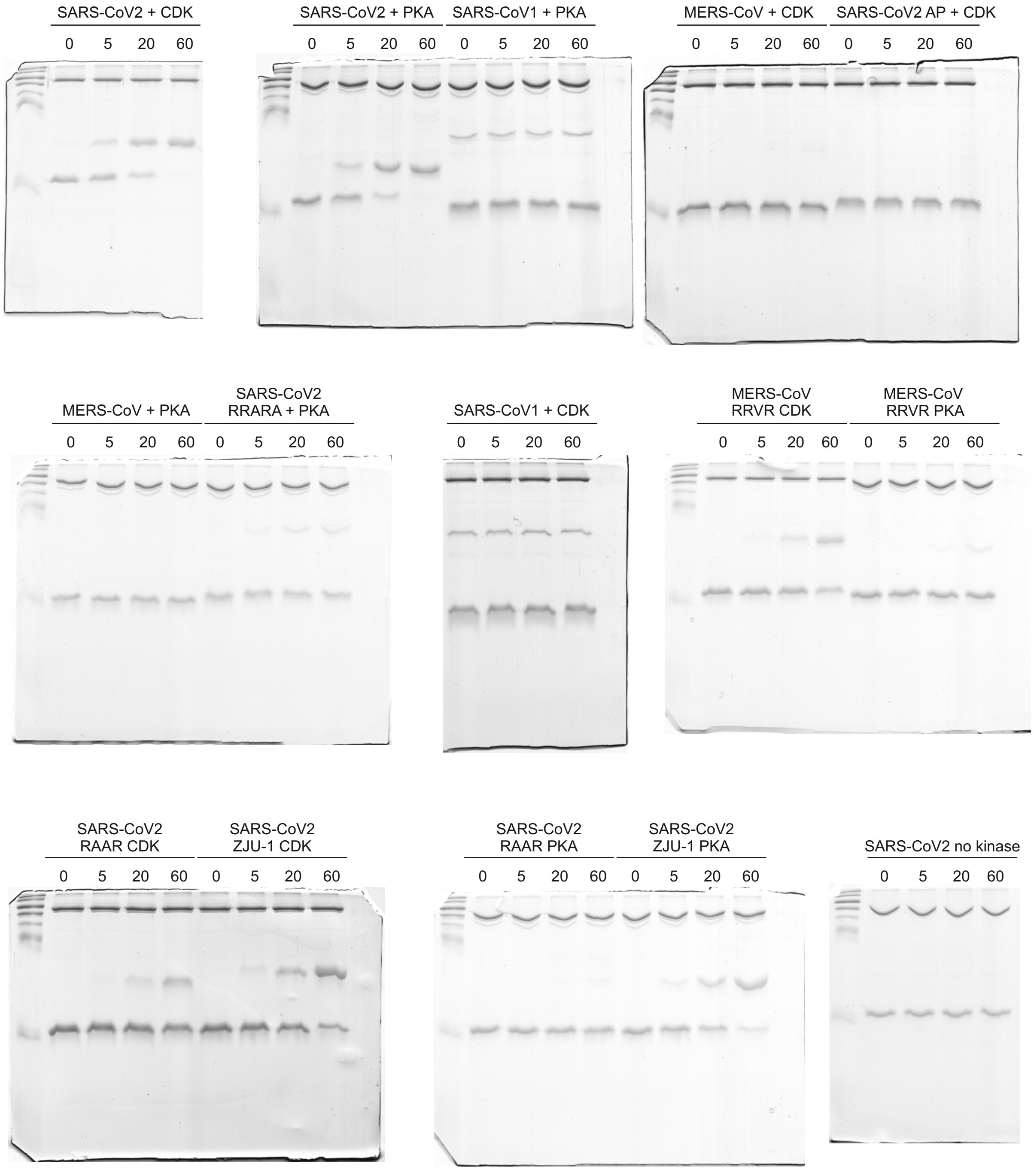

Supplementary Figure 3. Uncropped gels of Figure 3.

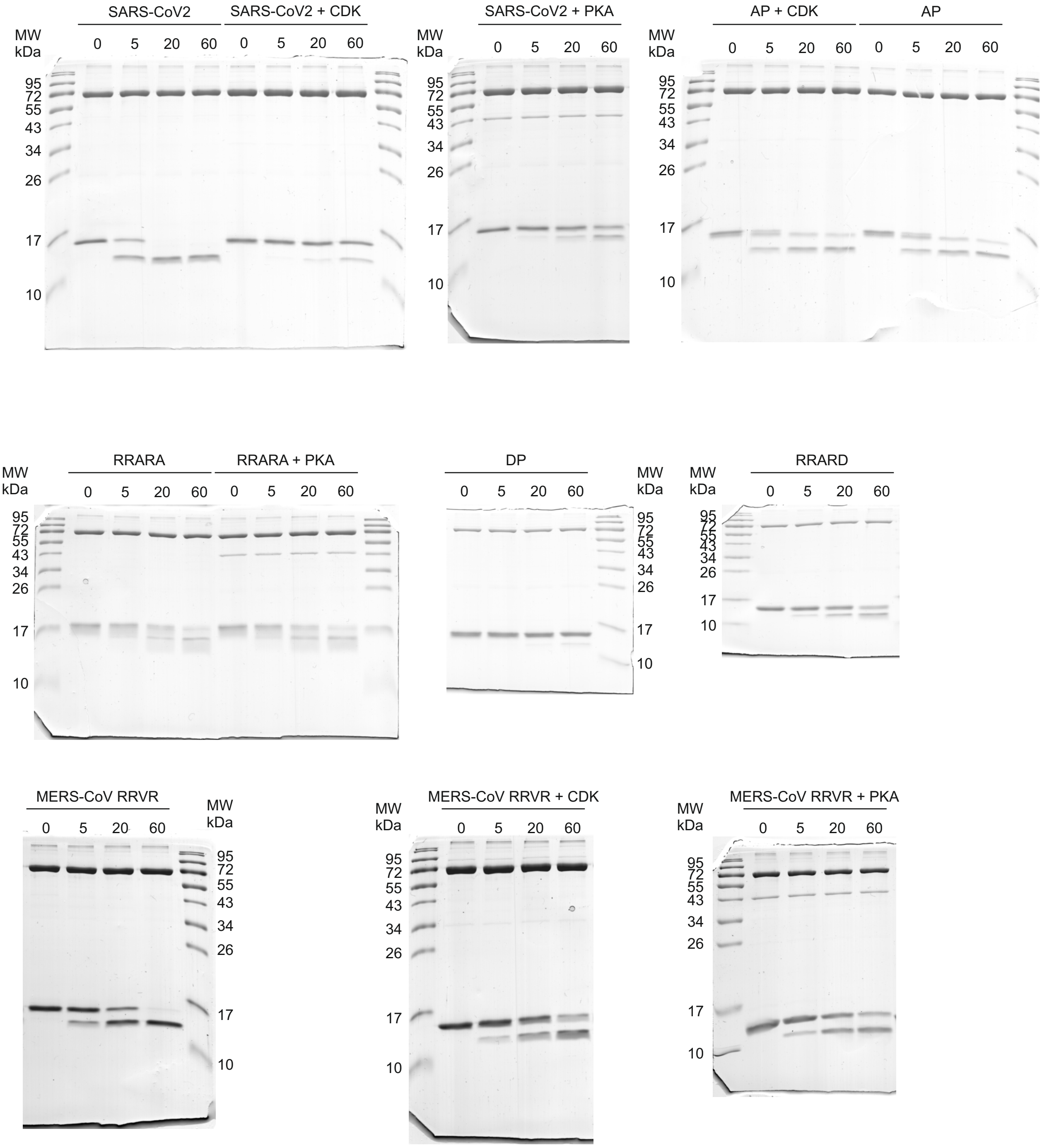

Supplementary Figure 4. Uncropped gels of Figure 4.
